# Consumer Preference for the Chevon Sausage in Blind and Nonblind Sensory Evaluations: A Comparative Study

**DOI:** 10.1155/2022/8736932

**Published:** 2022-07-29

**Authors:** Irene Rumbidzai Mazhangara, Eliton Chivandi, Ishmael Festus Jaja

**Affiliations:** ^1^Department of Livestock and Pasture Sciences, University of Fort Hare, Alice, South Africa; ^2^School of Physiology, University of Witwatersrand, Johannesburg, South Africa; ^3^Department of Agriculture and Animal Health, University of South Africa, Roodepoort Johannesburg 1710, South Africa

## Abstract

There are strong perceptions regarding chevon consumption, with its strong aroma and smell and its stringiness and gaminess being chief among them. Processing chevon into by-products has resolved this negative perception associated with fresh chevon. A blind and nonblind comparative sensory evaluation was performed to investigate participant preference for the chevon sausage versus pork and beef sausages. The sausages were made from minced shoulder meat. After grilling the sausages, they were cut into 0.5 cm thick slices. There were 52 and 20 participants in the blind and nonblind sensory evaluations, respectively. Using a 9-point hedonic scale, the participants evaluated each sausage for its juiciness, flavor, tenderness, and overall satisfaction. During the blind sensory evaluation, the participants were also asked to identify one of the most distinct sausages since the identity of the sausages was not known. The results showed no significant differences (*P* > 0.05) in the preferences for any of the sausages during the blind sensory evaluation. The choice for the most distinct sausage showed that pork (57.69%), beef (32.69%), and chevon (9.62%) sausages were all recognized. In the nonblind sensory, knowledge of the identity of the sausage significantly (*P* < 0.05) influenced South African participants' liking of the chevon sausage, with pork and chevon being the most and least liked sausages, respectively.

## 1. Introduction

Chevon is one of the most consumed red meats globally and is frequently included as a source of animal protein in everyday meals [1]. However, this may surprise most black, indigenous South Africans who usually only consume chevon at special traditional ceremonies [2, 3]. South Africans typically consume poultry, pork, mutton/lamb, and beef regularly [2, 4]. Among these meats, poultry and pork are the most consumed since they are relatively affordable to the general population [5]. Until recently, the price was a crucial factor in food choice. This has largely led to consumers purchasing more cost-effective meats [2]. However, due to increased nutritional awareness among consumers regarding the effects of poor diets, particularly the negative impacts on the metabolic health of high-calorie diets, there is a preference for more healthy food [6]. Consumer preference for leaner meat could serve as a vehicle to create a market for chevon.

Despite the nutritional profile that has a positive impact on consumer health, the consumption of chevon is still limited. There is considerable evidence of the taste and stringiness of chevon contributing to its lower demand [7]. Meat-derived products provide consumers with options and help to improve the shelf life of the products. Conventionally, meat products are derived from beef, pork, and mutton and little, if any, from chevon. As a result, there is a need to grow and develop a market for chevon-derived products and to ascertain whether consumers will accept them. Therefore, this study sought to find out how consumers liked the chevon sausage versus pork and beef sausages.

## 2. Materials and Methods

### 2.1. Collection of Meat Samples and Preparation of Sausages

5 kg of (chevon, beef, and pork) shoulder meat and 2 kg of (chevon, beef, and pork) fat were obtained from a high-throughput commercial abattoir in South Africa's Eastern Cape Province. The Boer goats (live weight range: 30-40 kg), Bonsmara cattle (live weight range: 450-500 kg), and Landrace pigs (live weight range: 310 to 400 kg) were used to produce the chevon, beef, and pork samples. After trimming off the visible fat and connective tissue, the meat samples were cut into small cubes, and each type of sausage was then prepared by mixing 80%, 20%, 2%, and 0.5% of the meat, fat, salt, and pepper, respectively. Each type of sausage mixture was then thoroughly mixed and minced with a 5 mm grinder (Torino, Italy: Trespade 22 EL Plus). Using a sausage filler machine (Torino, Italy: Trespade SFT0005), the mince was squeezed into natural sheep casings (Freddy Hirsch: 22 mm diameter). After that, the sausages were packed in polyamide-polythene bags and refrigerated at 4°C.

### 2.2. Proximate Analysis

The moisture content was determined by drying the sausages to a constant weight in an oven (100°C). The ash, fat, and protein content of the dried sausages was determined using the Association of Official Analytical Chemists [8] standard procedures: methods 978.04, 930.09, and 930.05, respectively.

### 2.3. Sensory Analyses

Informed consent was obtained from each participant. Seventy-two University of Fort Hare Animal Science Students formed an untrained consumer panel. Out of the total participant population, 52 participated in the blind and 20 in the nonblind analyses. Blind and nonblind sensory analyses of different types of sausages using a 9-point hedonic scale were performed to measure tenderness, juiciness, flavor, and overall satisfaction. In the blind sensory analysis, the participants were asked to identify one of the most distinct sausages since the identity of the sausages was not known. Participants were also asked to like each sausage (1 = most favorite (MF), 2 = next favorite (NF), or 3 = least favorite (LF)). The sausage samples were grilled until the internal temperature reached 75°C, then cut into about 0.5 cm thick pieces, labeled, and kept warm. Participants in the blind sensory evaluation tasted the three different sausages without being told which ones they were tasting. Before tasting each sausage, the participants were given unsalted crackers and water to refresh their palates. The nonblind survey had one exception: participants knew what kind of sausage they were evaluating.

### 2.4. Statistical Analysis

The data is presented as mean ± SD. For data analysis, the MINITAB 17 statistical package was used. ANOVA (one-way analysis of variance) was done. Tukey's Studentized Range Test was performed to separate the means. The significance level was set to *P* < 0.05. The dataset on the liking of the sausages was analyzed using Correspondence Analysis (CA) XLSTAT (2010).

## 3. Results

### 3.1. Proximate Composition of the Sausage Samples

The proximate composition (ash, fat, protein, and moisture) of the sausages (pork, beef, and chevon) is shown in Table 1.

### 3.2. Participant Demographics

Table 2 shows the demographic characteristics of the participants. The majority of the participants (62.55%) were female. South Africans made up the majority of participants (77.78%), while non-South Africans made up the minority (22.22%). Most participants who evaluated the sausages (76.54%) had tertiary education, while others had primary (12.76%) and secondary (10.70%) education.

### 3.3. Hedonic Evaluation of the Sausages in the Blind Sensory Analysis

Table 3 shows how much each of the three sausages was liked or disliked by the participants in terms of juiciness, flavor, tenderness, and overall satisfaction. The participants liked the tenderness, flavor, and juiciness of the chevon sausage. Importantly, the participants expressed overall satisfaction with the chevon sausage. There were no significant variations (*P* > 0.05) in the liking of pork and beef sausages between the two participant groups, but the non-South African participants liked the chevon sausage more (*P* < 0.05) than South Africans. There was no difference (*P* > 0.05) in how much the participants liked the flavor of the sausages.

**Figure 1:**
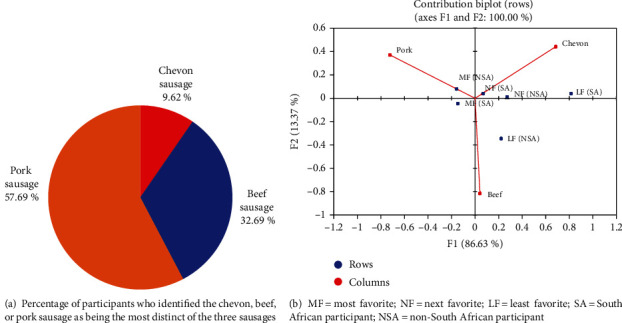
Percentage of participants who identified the chevon, beef, or pork sausage as being the most distinct of the three sausages (a). Contribution biplot representing the average liking of each sausage (b).

The choice between the three sausages in the blind sensory analysis was also evaluated. Participants were asked to name one of the most recognizable sausages as the identities of the sausages were unknown (Figure 1(a)). According to the findings, the majority of participants (57.69%) indicated that pork was the most distinctive, followed by beef (32.69%) and then chevon (9.62%).

The liking of each sausage is shown in Figure 1(b). The results showed that participants liked pork (61.54%) more, followed by beef (51.92%) and chevon (42.31%). In general, the results revealed that the majority of South African participants liked pork (56.41%) and beef sausages (51.28%). Non-South Africans, on the other hand, preferred the chevon sausage (53.85%) than among South Africans (38.46%).

### 3.4. Hedonic Evaluation of the Sausages: Comparisons between the Blind and Nonblind Sensory Evaluations

**Figure 2:**
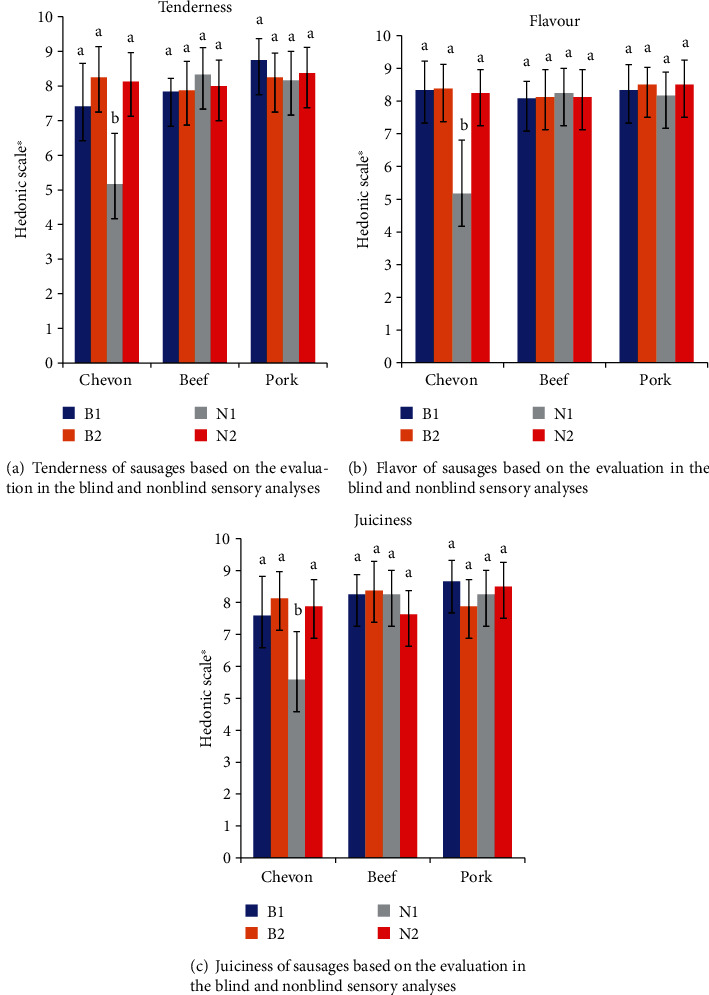
Tenderness (a), flavor (b), and juiciness (c) of sausages based on the evaluation in the blind and nonblind sensory analyses. ^a,b^Means that are different on the same sausage indicate significant differences (*P* < 0.05). B1 = South Africans in blind sensory analysis; B2 = non-South Africans in blind sensory analysis; N1 = South Africans in nonblind sensory analysis; N2 = non-South Africans in nonblind sensory analysis. ^∗^9-point hedonic scale: 1 = dislike extremely, 2 = dislike very much, 3 = dislike moderately, 4 = dislike slightly, 5 = neither dislike nor like, 6 = like slightly, 7 = like moderately, 8 = like very much, and 9 = like extremely.

**Figure 3:**
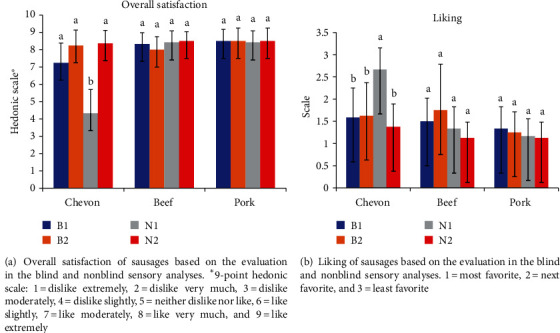
Overall satisfaction (a) and liking (b) of sausages based on the evaluation in the blind and nonblind sensory analyses. ^a,b^Means that are different on the same sausage indicate significant differences (*P* < 0.05). B1 = South Africans in blind sensory analysis; B2 = non-South Africans in blind sensory analysis; N1 = South Africans in nonblind sensory analysis; N2 = non-South Africans in nonblind sensory analysis.

Figures 2 and 3 show how much participants liked or disliked the tenderness, flavor, juiciness, and overall satisfaction of chevon, beef, and pork sausages in blind and nonblind sensory evaluations. During the blind sensory evaluation, no significant differences (*P* > 0.05) were observed in tenderness, flavor, juiciness, and overall satisfaction of all the sausages. However, the findings also revealed that a participant's liking of the chevon sausage was influenced by the sausage's identification. In terms of tenderness, flavor, juiciness, and overall satisfaction of the chevon sausage, there were significant differences (*P* < 0.05) between the blind and nonblind sensory evaluations by South African participants.

The liking of the three sausages is shown in Figure 3. During the blind sensory evaluation, there were no significant differences (*P* > 0.05) in the liking of all the sausages between the South African and non-South African consumers. However, there were significant differences (*P* < 0.05) in the sensory evaluations of chevon sausage liking between the blind and nonblind groups. In the blind sensory test, South African participants' preferences for the chevon sausage were slanted towards the next favorite (1.58), while chevon sausage liking was skewed towards the least favorite (2.67) during the nonblind sensory.

## 4. Discussion

Consumer sensory assessments reveal preferences and levels of acceptance for meat and meat products. Importantly, the consumer perceptions of the quality and acceptability of meat and meat products have a direct impact on the meat industry's profitability [9]. Participants in the blind sensory analysis tasted the three different sausages without being aware of their identities, which allowed them to assess the attributes of chevon sausages [7]. This approach is consistent with the assertion that panelists who are blind are more likely to provide objective sensory evaluations of the product [6]. Both the South African and non-South African participants positively perceived the chevon sausage according to the blind sensory data. However, there was no difference in the perception of flavor by the participants. These findings suggest that the two participating groups were unable to detect the distinguishing chevon flavor in the sausage, demonstrating the similarity of the sausages. It can be inferred that the sensory evaluation of the participant panel could not distinguish between the flavors of the different sausages. This could be taken to mean that the processing of chevon into sausages significantly reduces the distinct flavor of chevon.

Overall, the two participating groups liked every sausage to the same degree, although there were some differences in the liking of the chevon sausage. These observations suggest that the chevon sausage received favorable ratings across the board and was on par with beef and pork sausages. The current study's findings are in line with those of Jacques and Norwood [7], who reported favorable ratings for chevon, viz., tenderness, juiciness, flavor, and overall satisfaction. Given that the majority of the participants had never eaten any chevon-derived product before and that most of the South African participants were not accustomed to chevon, it was expected that the chevon sausage would be the most distinctive of the three sausages. The pork sausage was found to be the most distinct in the current investigation, which is similar to the findings reported by Jacques and Norwood [7]. In a blind study, they reported pork to be the most distinct in comparison to chevon and beef. As a result, the chevon sausage, which was unfamiliar to most participants, was consequently rated more favorable than pork. Despite the general frowning upon or poor acceptability of chevon and its products such as sausages, findings from the current study show that, in a blind evaluation, chevon sausages could be consumed without compromising the satisfaction with the overall eating quality. However, in the nonblind evaluation, South African participants had the least preference for chevon sausages, while their non-South African counterparts had a higher preference for chevon sausages. Other studies have shown that the South African Xhosa tribe tended to give chevon low sensory scores [10, 11]. The lack of chevon in the diets of most South Africans may be responsible for these results. Moreover, the consumption of chevon among South Africans is limited to specific cultural practices that occur rarely in any given year [2, 3]. Low exposure to any type of meat resulting from restricted encounters with specific meat has been reported to result in low consumer acceptability [12]. The low chevon sausage scores by South Africans could be inferred to be due to the infrequent exposure, while its high ranking among non-South Africans could be due to more frequent exposures [13, 14] and chevon experience.

The findings of the nonblind sensory evaluation showed that knowledge of the sausage identity had a negative impact on South African participants' liking of the chevon sausage. In comparison to the blind evaluation, where the South African participants liked the flavor of the chevon sausage very much, the nonblind evaluation showed a moderate liking of the tenderness and juiciness of the chevon sausage. These findings contradict those of Schouteten et al. [15], who discovered that under blind and nonblind sensory tests, insect-based burgers had a low overall liking and a considerably higher liking among young consumers. The primary determinant of product preference is thought to be the product's sensory quality [15]. It can, therefore, be inferred that the current study's findings confirm the chevon sausage's evident favorable sensory qualities. However, prior to evaluation, knowledge of the type of sausage compromised the liking scores of the chevon sausage. This knowledge-mediated decline in scores could have been influenced by the perceptions and beliefs of the South African participants.

The acceptance of the chevon sausage in the current study under blind evaluation reflects the acceptance and good performance of chevon in comparison to other red meats in the results of the blind studies published by Jacques and Norwood and Nelson et al. [7, 16]. Therefore, given that most of the study participants enjoyed eating the chevon sausage, this research supports its acceptability.

## 5. Conclusions

The study's findings, which were based on consumer preferences, show that the chevon sausage is acceptable and pleasing to most participants. In addition, the findings also show that a sizable segment of consumers exists who value the sensory characteristics of the chevon sausage. Given the findings of this study, it is apparent that the chevon sausage has a niche market. However, increased access to the products and understanding of chevon's health benefits could improve South African participants' relatively low liking of the chevon sausage in the nonblind sensory test. Therefore, there are opportunities to boost demand for chevon-derived products in order to satisfy the health-conscious consumers and consumers who enjoy the delicacy of this meat. Further research is recommended using a larger cross-country sample to obtain a true representative of the groups of consumers.

## Figures and Tables

**Table 1:** Proximate composition of beef, chevon, and pork sausages.

Parameter (%)	Sausage type		
	Beef	Chevon	Pork
Moisture	69.29 ± 0.877	72.90 ± 1.252	74.27 ± 1.322
Protein	25.60 ± 0.990	22.75 ± 0.071	25.92 ± 0.849
Total fat	10.60 ± 0.141	7.40 ± 0.424	15.35 ± 0.636
Ash	1.00 ± 0.042	1.01 ± 0.078	1.16 ± 0.007

Values expressed as mean ± SD, *n* = 2.

**Table 2:** Demographic data of participants.

Variable	Frequency	Percentage
Gender	25	34.72
Male		
Female	47	65.28
Age		
<20	3	4.17
20-25	27	37.50
26-30	26	36.11
31-40	14	19.44
41-50	2	2.78
Nationality		
South Africa	53	73.61
Nigeria	11	15.28
Zimbabwe	6	8.33
Uganda	1	1.39
Botswana	1	1.39

**Table 3:** Hedonic scores^∗^ for each sausage and attribute in blind sensory.

	Chevon sausage	Beef sausage	Pork sausage
	Tenderness		
SA	7.46^bA^ ± 0.94	8.05^aA^ ± 0.86	8.44^aA^ ± 0.75
NSA	8.23^aB^ ± 0.83	8.08^aA^ ± 0.76	8.23^aA^ ± 0.73
	Flavor		
SA	7.82^aA^ ± 1.02	8.13^aA^ ± 0.86	8.28^aA^ ± 0.69
NSA	8.31^aA^ ± 0.75	8.39^aA^ ± 0.77	8.15^aA^ ± 0.80
	Juiciness		
SA	7.56^bA^ ± 1.10	8.31^aA^ ± 0.73	8.36^aA^ ± 0.74
NSA	8.31^aB^ ± 0.86	8.39^aA^ ± 0.77	8.15^aA^ ± 0.80
	Overall satisfaction		
SA	7.39^bA^ ± 1.04	8.18^aA^ ± 0.79	8.46^aA^ ± 0.68
NSA	8.23^aB^ ± 0.83	8.15^aA^ ± 0.80	8.46^aA^ ± 0.66

^∗^9-point hedonic scale: 1 = dislike extremely, 2 = dislike very much, 3 = dislike moderately, 4 = dislike slightly, 5 = neither dislike nor like, 6 = like slightly, 7 = like moderately, 8 = like very much, and 9 = like extremely. SA = South African participant; NSA = non-South African participant. ^a,b^Mean with superscripts across a row indicates significant differences (*P* < 0.05). ^A,B^Mean with superscripts within a column indicates significant differences (*P* < 0.05).

## Data Availability

All data generated or analyzed during this study are included in this published article.

## References

[1] Mazhangara I. R., Chivandi E., Mupangwa J. F., Muchenje V. (2019). The potential of goat meat in the red meat industry. *Sustainability*.

[2] Erasmus S. W., Hoffman L. C. (2017). What is meat in South Africa?. *Animal Frontiers*.

[3] DAFF (2018). A profile of the South African goat market value chain 2018, Department of Agriculture, Forestry and Fisheries.

[4] (2015). *The South African meat report 2015*. *Pretoria*: *Department of Agriculture*, *Forestry and Fisheries*.

[5] NAMC (National Agricultural Marketing Council) (2016). Media release: food price monitor November 2016. *NAMC*.

[6] Damaziak K., Stelmasiak A., Riedel J. (2019). Sensory evaluation of poultry meat: a comparative survey of results from normal sighted and blind people. *PLoS One*.

[7] Jacques K., Norwood F. (2017). Consumer preference for goat meat in a blind sensory analysis. *Sheep & Goat Research Journal*.

[8] AOAC (2005). *Official Methods of Analysis of the Association of Official Analytical Chemists International*.

[9] Ristić M., Troeger K., Đinović-Stojanović J., Knežević N., Damnjanović M. (2017). Consumer perception and acceptance of pork and chicken sausage. *IOP Conference Series: Earth and Environmental Science*.

[10] Moyo B., Masika P., Muchenje V. (2014). Effect of feeding moringa (Moringa oleifera) leaf meal on the physico-chemical characteristics and sensory properties of goat meat. *South African Journal of Animal Science*.

[11] Xazela N. M., Chimonyo M., Muchenje V., Marume U. (2011). Consumer sensory evaluation of meat from South African goat genotypes fed on a dietary supplement. *African Journal of Biotechnology*.

[12] Borgogno M., Corazzin M., Saccà E., Bovolenta S., Piasentier E. (2015). Influence of familiarity with goat meat on liking and preference for capretto and chevon. *Meat Science*.

[13] Juma G. P., Ngigi M., Baltenweck I., Drucker A. G. (2010). Consumer demand for sheep and goat meat in Kenya. *Small Ruminant Research*.

[14] Lawal-Adebowale O. A. (2012). Dynamics of ruminant livestock management in the context of the Nigerian agricultural system. *Livestock Production*.

[15] Schouteten J. J., De Steur H., De Pelsmaeker S. (2016). Emotional and sensory profiling of insect-, plant- and meat-based burgers under blind, expected and informed conditions. *Food Quality and Preference*.

[16] Nelson M. C., Whitehead J., Mobini S., Brown N. B., Thomas M. (2004). Segmenting niche goat-meat markets. *Journal of Food Distribution Research*.

